# Unique sequelae of portal vein thrombosis in a pediatric patient with cystic echinococcosis: A case report

**DOI:** 10.1002/jpr3.12066

**Published:** 2024-03-19

**Authors:** Steven Lin, Terry C. Dixon, Hamza Hassan Khan, Martha M. Munden, Janaina N. Anderson

**Affiliations:** ^1^ College of Medicine Medical University of South Carolina Charleston South Carolina USA; ^2^ Division of Pediatric Infectious Diseases, Department of Pediatrics Medical University of South Carolina Charleston South Carolina USA; ^3^ Division of Pediatric Gastroenterology, Department of Pediatrics Medical University of South Carolina Charleston South Carolina USA; ^4^ Division of Pediatric Diagnostic Radiology, Department of Diagnostic Radiology Medical University of South Carolina Charleston South Carolina USA

**Keywords:** albendazole, hematochezia, hydatid cyst, mesenteric venous thrombosis, PAIR procedure

## Abstract

This case report presents a rare complication of hepatic cystic echinococcosis in a 12‐year‐old Latino male, residing in a nonendemic region, who developed long‐term sequelae of portal vein thrombosis accompanied by the emergence of a hyper‐vascular sigmoid colon mass. Portal vein involvement in hepatic cystic echinococcosis is exceedingly uncommon, with limited documented cases. The presentation of the patient included intermittent hematochezia, abdominal pain, and fatigue. Imaging revealed liver cysts and chronic portal vein thrombosis with cavernous transformation, resulting in portal hypertension. Notably, the patient also exhibited mesenteric venous thrombosis, further complicating the clinical picture. The diagnosis was confirmed through echinococcus serology testing. Treatment involved a six month course of Albendazole, puncture‐aspiration‐injection‐reaspiration procedure, splenectomy, and splenorenal shunt to alleviate portal hypertension. This case underscores the significance of considering portal hypertension secondary to hepatic cystic echinococcosis, even in nonendemic regions, particularly in pediatric patients with unique clinical presentations.

## 1 INTRODUCTION

*Echinococcus granulosus* is a zoonotic infection caused by the metacestode stage of tapeworms of the genus *Echinococcus*. Although echinococcosis is considered rare in the United States, most cases are imported from individuals immigrating from endemic regions. While symptoms depend on the organs affected, the clinical presentation is frequently nonspecific and some patients tend to be paucisymptomatic. Liver involvement occurs in approximately 50%–77% of cases and can manifest as the development of hydatid cysts.^1^ In this case report, we aim to present a rare complication of hepatic cystic echinococcosis (CE), in which the patient developed long‐term sequelae of portal vein thrombosis (PVT) accompanied by emergence of a hyper‐vascular sigmoid colon mass. Portal vein involvement is exceedingly uncommon, and to our knowledge, this is the first reported case in a pediatric patient treated in North America.

## 2 CASE REPORT

A 12‐year‐old Latino male presented with a six month history of intermittent hematochezia and fatigue. Prior to admission, he also endorsed abdominal pain and chronic constipation for the past nine months, with multiple visits to his primary care provider. Symptoms abruptly transitioned to diarrhea one week prior to seeking medical attention. His medical history revealed a previous episode of eosinophilia which has since resolved, characterized by elevated eosinophil percentage (29.6%) and absolute eosinophil count (4700 µL), during an emergency department visit three years prior due to abdominal pain. He had traveled to Guatemala several years ago. Family history was unremarkable for coagulopathies, inflammatory bowel diseases, or polyps. The patient was born at term with no gestational complications and no history of umbilical vein catheterization.

A complete blood count was ordered which showed pancytopenia with hemoglobin at 5.9 g/dL; white blood cell count at 3.7 K/µL; and platelet count at 117 K/µL. The differential was: neutrophils 54%, lymphocytes 32.1%, monocytes 10.7%, eosinophils 2.4%, and basophils 0.5%. γ‐glutamyl transferase value was elevated (36 U/L), otherwise the remaining laboratory tests were unremarkable. Thrombophilia workup for protein C and S deficiency, factor V Leiden, prothrombin G20120A, antiphospholipid antibody, and paroxysmal nocturnal hemoglobinuria all resulted within normal limits.

**Figure 1:**
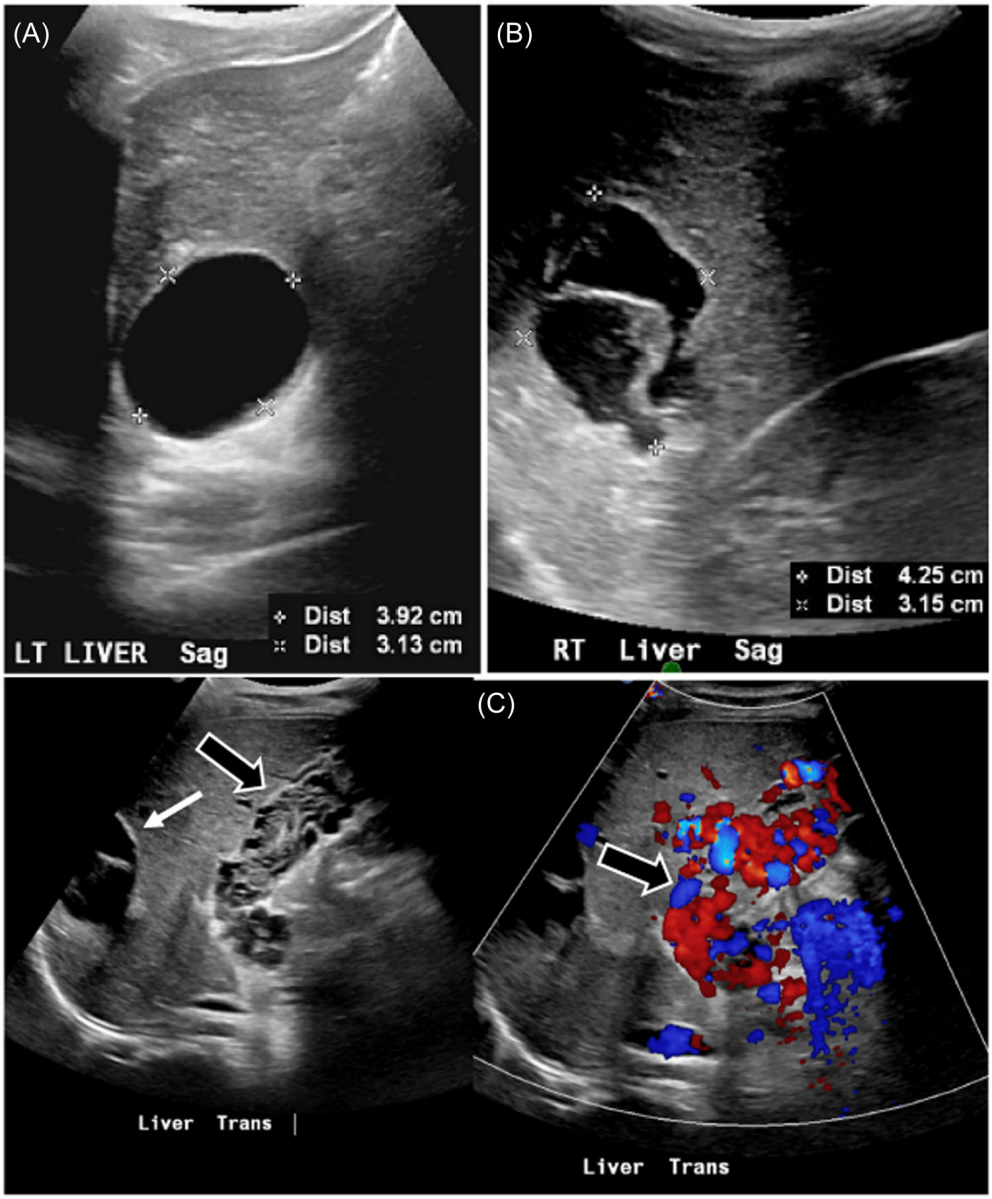
(A) Liver US image that revealed a unilocular, anechoic cystic lesion in segment II measuring 3.9 × 3.1 cm. (B) Liver US image that revealed “water lily” sign in cystic lesion in segment VI/VII measuring 4.2 × 3.1 cm. (C) Gray scale and color doppler US images show striking varices within gallbladder wall (thick arrows) as well as a portion of the cystic lesion in the right liver (thin arrow).

**Figure 2:**
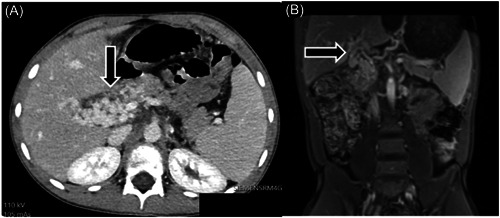
(A) Axial CT image of extensive varices at the porta hepatis. (B) Coronal T1 fat‐saturated postcontrast MRI showing massive varices at the porta hepatis.

**Figure 3:**
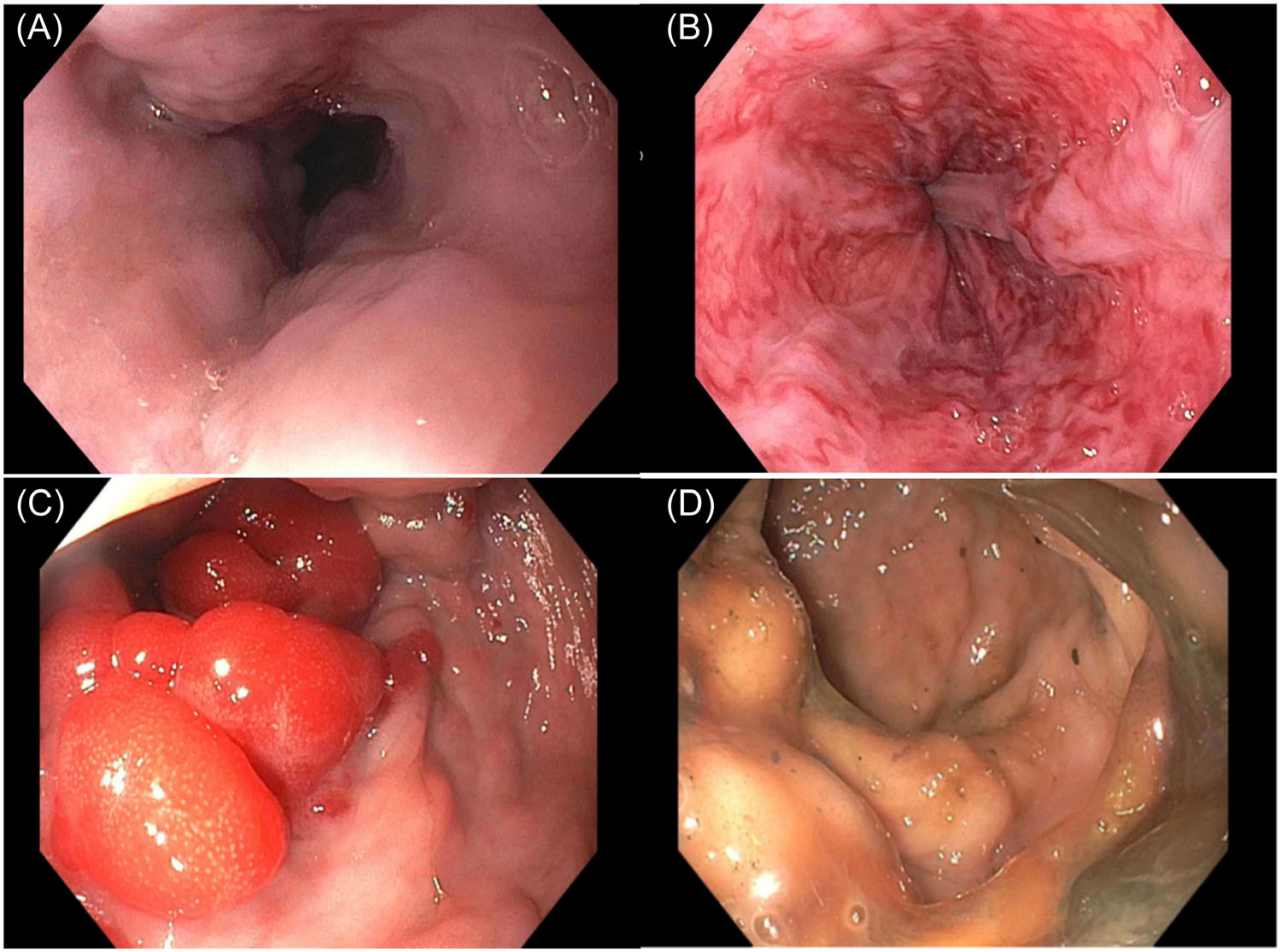
(A) Endoscopic image of the middle third of the esophagus reveals grade III varices. (B) Vascular ectasias found in the lower third of the esophagus. (C) Large vascular lesions noted in the sigmoid colon. (D) Prominent veins seen in the rectum.

Given the history of lower gastrointestinal bleeding, male gender, and the age of the patient, with low clinical suspicion of gastroenteritis, a Meckel scintigraphy scan was ordered, revealing abnormal tracer uptake in the lower left quadrant (LLQ). Subsequently, an abdominal ultrasound (US) was obtained, demonstrating left colonic wall thickening with hyperemia and ectatic vascular structures in the right upper quadrant abutting the liver (Figure 1). To better characterize the findings, a computed tomography (CT) scan of the abdomen was performed. The CT scan revealed a simple cyst in liver segment II measuring 3.6 cm and a cystic lesion in liver segments VI/VII measuring 4.4 cm with internal layering debris. Additionally, the scan demonstrated sequelae of chronic PVT with cavernous transformation at the porta hepatis (Figure 2). Noteworthy associated findings included splenomegaly, grade III esophageal varices, small volume ascites, and severe long segment thickening of the descending colonic wall, accompanied by mural edema (Figure 3). The decision was made to perform a US‐guided percutaneous non‐targeted liver biopsy to assess for parenchymal hepatic diseases. Biopsy results showed benign liver parenchyma with minimal lobular neutrophilic inflammation and absence of fibrosis.

Due to the past travel history, history of eosinophilia, and hepatic cystic lesions, echinococcus serology test was ordered which revealed positive IgM antibodies for *E. granulosus*. A diagnosis of chronic PVT with cavernous transformation secondary to liver hydatid disease was made. The hydatid cysts were classified as WHO stage CE1 and CE3a.

A six month course of Albendazole (400 mg/day) was prescribed and a puncture‐aspiration‐injection‐reaspiration (PAIR) procedure was performed. In addition, meningococcal and pneumococcal vaccinations were administered in anticipation for a splenectomy and proximal splenorenal shunt to decompress the portal vasculature. The preference for a splenorenal shunt over alterative portosystemic shunts, such as a meso‐rex bypass, aimed to prevent inadvertent puncture and rupture of the hydatid cysts. Additionally, considering the challenging anatomy of the splenic vein and the tail of the pancreas, the decision was made to perform a proximal splenorenal shunt.

At his six month follow‐up visit, the patient denied fatigue, abdominal pain, and diarrhea, but he continued to endorse hematochezia, which was attributed to his constipation. Abdominal US of both hydatid cysts showed decreased sizes with increased internal complexity. Although our patient endorsed reduced symptomatic burden, imaging continued to demonstrate persistent cavernous transformation at the porta hepatis, and further follow‐ups are necessary to monitor for changes in the collateral vessels.

## 3 DISCUSSION

*E. granulosus* is endemic worldwide but most prevalent in China, Central Asia, South America, North and East Africa, Australia, and Mediterranean and Eastern European countries due to its life cycle involving canids and ruminants.^2^ Humans are incidental intermediate hosts and can be exposed to parasitic eggs by ingesting contaminants containing infected canid stool. The eggs release oncospheres that penetrate intestinal walls and migrate via bloodstream or lymphatic vessels to any anatomic location. While the liver is affected in most cases, the lungs are the second most common site of infection (15%–47%).^3^ Other less commonly affected organs include the spleen, kidneys, heart, central nervous system, bone, and several other regions.

The pathogenesis of PVT and cavernous transformation can be influenced by cirrhosis, infections, hypercoagulable states, and in rare instances, hydatid cystic disease. Although the exact mechanism of the development of portal hypertension secondary to CE is unknown, it is possible that the direct compression of the portal vein by the cyst can occur, resulting in mechanical obstruction and extrahepatic portal vein obstruction. This obstruction leads to increased portal pressure and subsequent portal hypertension. In response to the increased pressure, the liver undergoes vascular remodeling, giving rise to cavernous transformation of the portal vein. This phenomenon represents the compensatory mechanism of the liver to reroute blood flow and bypass the obstructed portal vein. Consequently, the elevated portal pressure affects the downstream portosystemic collaterals, leading to the development of collateral vessels such as esophageal, gastric, and rectal varices. The hydatid cyst in our patient was located in the periphery with radiographic images demonstrating patent hepatic veins which made this pathogenic mechanism less plausible.

**Figure 4:**
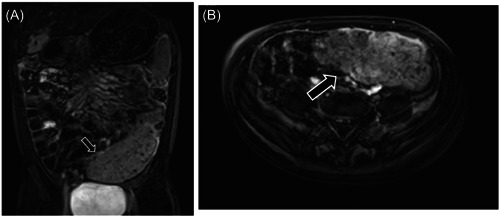
(A) MRI enterography image revealed a hyper‐vascular mass in the area of the sigmoid colon. (B) Transverse view of the sigmoid colon mass.

Notably, our patient exhibited an additional unique finding related to the downstream consequences of PVT. Magnetic resonance imaging (MRI) revealed marked thickening of the sigmoid wall and surrounding mesentery, accompanied by ectatic veins (Figure 4). This finding suggests the presence of secondary mesenteric venous thrombosis (MVT) in our patient. Secondary MVT is a condition where an underlying disorder induces a state of thrombophilia, venous stasis, and vascular injury within the bowel which all contribute to the formation of a thrombus.^4^ Although the pathophysiological association between PVT and MVT are not clear, increased venous pressures within the sigmoid vein, a branch of the mesenteric venous system, can cause profound bowel wall edema. Due to the extensive presence of collateral veins surrounding the sigmoid colon, chronic MVT should be suspected. Bleeding from sigmoid varices secondary to increased venous pressure was presumed to be the etiology of hematochezia in our patient. Moreover, studies have also demonstrated that a state of hypereosinophilia is associated with venous thromboembolism.^5^

Mainstay treatment for echinococcosis depends on the WHO staging and size of the cyst. In unilocular anechoic cystic lesion (CE1) less than 5 cm, Albendazole alone is the preferred treatment.^6^ However, in cases where albendazole treatment is not feasible or effective, a PAIR procedure can serve as an appropriate alternative approach. The PAIR procedure involves percutaneous puncture of the cyst, followed by aspiration of its contents, injection of scolicidal agents, and re‐aspiration to reduce the cyst size and improve patient outcomes. Albendazole inhibits microtubule assembly resulting in impaired glucose absorption which eventually leads to apoptosis. Outcomes in pharmacological management are variable depending on host characteristics, cyst size and age, and location. A comparative trial demonstrated that combining Albendazole with the PAIR procedure resulted in a significant reduction in cyst size.^7^ However, stage CE2 and CE3 hydatid cysts are most likely to relapse after PAIR procedure due to its compartmentalization.

PVT and cavernous transformation secondary to hepatic CE is an exceedingly rare occurrence, with only a limited number of documented cases in medical literature. To demonstrate the infrequency of this occurrence, a large Spanish study followed a cohort of 506 patients with CE for 20 years with only two‐patient recorded incidences of portal hypertension reported.^8^ To our knowledge, this case report represents the first documented instance of this unique phenomenon in a pediatric patient receiving treatment in North America. Our case highlights an unusual presentation of CE and the significance of considering portal hypertension secondary to hepatic CE especially in pediatric patients residing in nonendemic regions.

## CONFLICT OF INTEREST STATEMENT

The authors declare no conflict of interest.

## ETHICS STATEMENT

Ethical approval was waived by the local Ethics Committee of the Medical University of South Carolina in view of the nature of the study. Verbal and written consent were obtained from the patient and father to attain consent to publish this case report. All identifying information has been removed.
